# Supplementary material for: Deletion of wfs1 Impairs Oligodendrocyte Precursor Cells Dorsal Distribution and Myelination Through the wfs1-hmgcs1 Axis in Zebrafish
Source: Biology (Basel). 2026 Aug 16;15(16):1402. doi: 10.3390/biology15161402 (PMC13510151; doi:10.3390/biology15161402)
Supplement: Supplementary file 1 [file biology-15-01402-s001.zip › File S1-Transcriptome sequencing data and differentially expressed genes (DEGs) in wfs1-- zebrafish at 5 dpf/2.KEGG/wfs1KOvsWT/DOWN/src/dre00310.html]

dre00310


- dre:368262

- Down regulated genes

368262(-1.10235845855677)

- dre:393860

- Down regulated genes

393860\_1(-1.09876656700232)

- dre:100005587

- Down regulated genes

100005587\_1(-1.54424530950344)
- dre:100006238

- Down regulated genes

100006238\_1(-1.31531604695891)

- dre:556535

- Down regulated genes

556535\_1(-1.71366832530581)
- dre:100003615

- Down regulated genes

100003615\_1(-5.97421248289227)

- dre:556535

- Down regulated genes

556535\_1(-1.71366832530581)
- dre:100003615

- Down regulated genes

100003615\_1(-5.97421248289227)

- dre:556535

- Down regulated genes

556535\_1(-1.71366832530581)
- dre:100003615

- Down regulated genes

100003615\_1(-5.97421248289227)

- dre:100005587

- Down regulated genes

100005587\_1(-1.54424530950344)
- dre:100536795

- Down regulated genes

100536795(-1.64304185838866)
- dre:100006238

- Down regulated genes

100006238\_1(-1.31531604695891)

Close
